# Effects of prohexadione calcium spraying during the booting stage on panicle traits, yield, and related physiological characteristics of rice under salt stress

**DOI:** 10.7717/peerj.14673

**Published:** 2023-01-23

**Authors:** XiXin Huang, Dianfeng Zheng, Naijie Feng, Anqi Huang, Rongjun Zhang, Fengyan Meng, Yin Jie, Baomin Mu, Dewei Mu, Hang Zhou

**Affiliations:** 1 Guangdong Ocean University, College of Coastal Agriculture Sciences, Zhanjiang, Guangdong, China; 2 South China Center of National Saline-tolerant Rice Technology Innovation Center, Zhanjiang, Guangdong, China; 3 Shenzhen Research Institute of Guangdong Ocean University, Shenzhen, Guangdong, China

**Keywords:** Prohexadione calcium, Salt stress, Rice, Booting stage, Panicle traits

## Abstract

Prohexadione calcium (Pro-Ca), as a growth retardant, can effectively alleviate the damage of salt stress to plants. In order to explore the effects of NaCl stress on the physiological characteristics and panicle traits of rice plants as well as the alleviating effect of Pro-Ca at the booting stage, we performed pot experiments on two rice cultivars: conventional rice ‘*Huanghuazhan*’ and hybrid rice ‘*Xiangliangyou900*’. Rice plants were treated with 0.3% NaCl 48 hours after Pro-Ca (100 mg L^−1^) treatment to study the effects of Pro-Ca on the physiological characteristics of the leaves and panicles, as well as the panicle and yield traits of rice under salt stress. Our analysis indicated that NaCl treatment inhibited the morphological growth parameters and photosynthetic efficiency, destroyed the antioxidant defense systems of leaves and panicles, increased soluble protein and proline in both rice cultivars. Foliar application of Pro-Ca significantly increased the leaf area, uppermost internode length, panicle length, panicle weight, number of primary branches, number of grains per panicle, seed setting rate and yield under salt stress. Pro-Ca application significantly affected chlorophyll content, net photosynthetic rate (Pn), stomatal conductance (Gs), transpiration rate (Tr), and apparent mesophyll conductance (AMC) in NaCl-treated rice cultivars compared with NaCl treatment alone. Moreover, Pro-Ca also increased ascorbic acid (AsA) content, enhanced superoxide dismutase (SOD), peroxidase (POD), catalase (CAT), and ascorbate peroxidase (APX) activity, and further increased the accumulation of soluble protein and proline in leaves and panicles. These results illustrated that foliar application of Pro-Ca at the booting stage could alleviate the damage caused by NaCl stress by regulating the physiological and metabolic processes of rice plants, thereby enhancing the stress resistance of the plants, increasing total rice yield in salt stress conditions.

## Introduction

The plant growth, development and yield are negatively affecting by a series of environmental stresses such as high temperature, chilling, drought and salinity. Salinity stress is one of the most significant environmental stresses a plant can face (FAO, 2019). Salinity leads osmotic and ionic stresses that results in the excessive production of reactive oxygen species (ROS) in plant cell, disruption of normal cellular metabolism, impairing various physiological functions and inhibits cellular components in plants (Arif et al., 2020; Gupta & Huang, 2014). According to one study, the total area of saline-alkali land in China has reached 99.13 million hm^2^ (Zhang & Wang, 2021), including many tidal flats. This is likely to increase as seawater intrusion leads to severe soil salinization (Yuvaraj et al., 2021). Salinity stress has significantly inhibited both ecological balance of the area and crop yield.

Rice (*Oryza sativa L.*) is a staple global crop that feeds approximately half of the world’s population (Singh et al., 2019) and is considered a salt-sensitive crop (Arif et al., 2019). Salinity inhibits the growth and development of rice plants (Chang et al., 2019), by affecting the morph-physiological traits of the plants, resulting in decreased grain yield (Nasrudin, Isnaeni & Fahmi, 2022). Improving the salt tolerance of rice plants will increase the development and utilization potential of saline-alkali land, which is conducive to sustainable agricultural development. The adverse effect of salt stress affects each growth and development stage of the rice (Fraga et al., 2010). The booting stage in rice plants is the transition period from vegetative growth to reproductive growth. It is the critical period for determining panicle traits, seed setting rate, and is the most sensitive period to the external environment (Wang et al., 2020). Previous studies showed that salinity adversely effects the plant development are more profound during the reproductive stage of rice such as differentiation of rice branches, leading to spikelet degeneration, the number of seeds, the seed setting rate, 1,000-grain weight and finally the grain yield of rice (Fraga et al., 2010; Mojakkir et al., 2015; Chakraborty et al., 2019). Therefore, it is necessary to specifically understand the response of rice to salt stress during the booting stage to lay a foundation for further research and also to metigate the salt tolerance of rice during this critical growth stage.

Chemical control is one way of alleviating salt stress to increase crop yield. It has the benefits of convenience, micro-efficiency, and speed. Many plant growth regulators successfully improved the salt tolerance of plants by regulating different physiological mechanisms (Qiu et al., 2014; Wei et al., 2017; Khan et al., 2021; Ali et al., 2021). As a new plant growth retardant, prohexadione calcium (Pro-Ca) has no residual toxicity to rotation crops and considered to be environmentally friendly compared to triazole retarders etc (Na et al., 2011; Soleimani Aghdam, 2013). Soleimani Aghdam (2013) showed that tomato plants after Pro-Ca treatment were able to maintain a higher proline level and had better membrane integrity to mitigate the adverse effects of chilling damage. Ozbay & Susluoglu (2016) used Pro-Ca as an initiator and found that it could modify some components in pepper seeds to improve their tolerance to cold stress. Feng et al. (2021) reported that foliar application of exogenous Pro-Ca could alleviate salt stress damage to soybean seedlings by improving photosynthesis, stimulating the antioxidant defense system, and increasing osmotic regulation. Although previous studies have shown that exogenous Pro-Ca has a regulatory effect on plants under salt stress conditions. These studies have been conducted mainly at the seedling stage of plants, and the effect of exogenous Pro-Ca on rice at the booting stage under salt stress has not been identified. Therefore, in this study, a pot experiment was conducted to study the effects of NaCl stress and the alleviation effect of Pro-Ca against NaCl stress on the agronomic, physiological, and yield traits of rice plants. This study aims to explore the mechanisms of Pro-Ca salinity stress alleviation in rice plants.

## Materials & Methods

### Plant material and growth conditions

The seeds of two rice cultivars—one conventional rice variety, ‘*Huanghuazhan*’ (HHZ), one hybrid rice variety, ‘*Xiangliangyou900*’ (XLY900)—were purchased from Hunan Longping Seed Industry Co., Ltd. and Hunan Nianfeng Seed Industry Technology Co., Ltd., respectively. The test reagent was 5% prohexadione calcium (Pro-Ca), provided by the Chemical Control Laboratory of College of Coastal Agriculture Sciences of Guangdong Ocean University.

The experiment was conducted in a greenhouse under the natural conditions, with day/night temperatures of 30/28 ± 2 °C. Rice seeds were disinfected with a 2.5% NaClO solution for 15 min and then rinsed thoroughly with distilled water. The seeds were then soaked in distilled water for 48 h in darkness at 30 °C. The seeds were sown in plastic floppy disks (54 cm × 27 cm × 6 cm) filled with red brick loam and nutrient soil (3:1, v/v) for seedling cultivation. The uniform size seedlings at the four-leaf stage were transferred into black plastic pots (upper inner diameter 31.5 cm × bottom diameter 22.5 cm × height 29.5 cm) with 15 kg of red brick loam as the base soil (five plants per pot).

A total of 2.03 g of nitrogen fertilizer (urea, converted to pure nitrogen), 0.9 g of phosphate fertilizer (di-ammonium phosphate, converted to P_2_O_5_), and 1.12 g of potassium fertilizer (potassium chloride, converted to K_2_O) were applied to each plastic pot in the following period. Nitrogen fertilizer was applied according to the following ratio: base fertilizer: tiller fertilizer: panicle fertilizer = 4:3:3, all phosphorus fertilizer was used as base fertilizer, and potassium fertilizer was applied according to the ratio: base fertilizer: panicle fertilizer = 7:3.

### Pro-Ca and salinity treatments

After transplanting, the main stem of each potted seedling was marked, and each pot maintained 1–2 cm of water on top. During the booting stage (77 days after sowing), 100 mg L^−1^ of Pro-Ca foliar spray was applied, and distilled water was used as the control. Each pot was only sprayed once with the same amount of either distilled water (control) or Pro-Ca (treatment). The concentration of Pro-Ca (100 mg L^−1^) was selected based on the results of our previous studies where different concentrations of Pro-Ca (25, 50, 75, 100, 125, 150 mg L^−1^) applied to rice showed varying results, with the clearest results observed with a Pro-Ca concentration of 100 mg L^−1^ (Yao et al., 2021). The Pro-Ca treatment was applied between 16:00–18:00 P.M. In order to make the electrical conductivity (EC) of the soil reach 3dS/m, 0.3% NaCl was added to each pot in the two salt treatment groups 48 h after the Pro-Ca was sprayed (percentage of soil salt content = dry mass of NaCl/dry soil mass). EC was measured with an EC sensor (shunkedaTR-6D, CHINA) at a depth of seven cm in the growing soil from treatment to harvest. The salt concentration used in this study was selected based on our preliminary experiments (Chen et al., 2022) and on previous reports (Efisue & Dike, 2020) which showed that rice is a salt sensitive crop with a threshold level of 3dS/m. The experiment was carried out in a completely randomized design, and each rice variety had four treatment groups: (1) control, distilled water (CK); (2) 0.3% NaCl (NaCl); (3) Pro-Ca + 0.3% NaCl (Pro-Ca + NaCl); and (4) Pro-Ca (Pro-Ca). Each treatment was tested with four replicates. After 14 days of NaCl treatment, the rice plants were harvested. The plant morphology and physiological indicators of the leaves and panicles were then determined. The collected plant samples were quickly frozen in liquid nitrogen and stored at −40 °C. The panicle and seed traits were analysed at full plant maturity.

### Growth parameters

Plant height was measured during the heading growth stage (14d after the NaCl treatment) from the soil surface to the marked, central stem sword leaf. The length and width of the sword leaf were also measured. The area of the sword leaf was calculated according to the following formula (Wei et al., 2020): leaf area = (length × width × 0.75). The dry weight of the shoot was also measured.

### Chlorophyll and gas exchange

The second functional leaf of the main stem (from the top of the plant) was used to measure the chlorophyll and gas exchange parameters. A SPAD-502 chlorophyll meter was used to measure the SPAD values of the upper, middle, and lower parts of the leaf, with the average value used as the final SPAD value. Photosynthetic parameters, including net photosynthetic rate (Pn), stomatal conductance (Gs), intracellular CO_2_ concentrations (Ci), and transpiration rate (Tr), were determined using the LI-6400 portable photosynthesis system (LI-6400, LI-COR, USA) between 9:00-11:30 A.M. The apparent mesophyll conductance (AMC) was estimated using the rate of Pn/Ci (Liu et al., 2012). The conditions in the leaf chamber were: photosynthetically active radiation (PAR) of 1,000 µmol m^−2^ s^−1^, CO_2_ concentration of 400 µmol mol^−1^, and leaf temperature of 26.0 °C. The data were automatically collected every three minutes.

### Determination of antioxidant and osmotic adjustment substances

PBS (phosphate buffer solution, pH 7.8, 50 mM) was pre-cooled on ice, and then used to homogenize the samples of fresh leaves (0.5 g) and panicles (0.5 g). After centrifugation (10,000 g/10 minutes/4 °C), the supernatant was collected to determine enzymatic activity. Superoxide dismutase (SOD) activity was measured spectrophotometrically at 560 nm to determine the photochemical reduction of nitro blue tetrazolium (NBT; Liu et al., 2018). One unit of SOD activity was defined as the amount of enzyme required to produce a 50% inhibition of the reduction of NBT. Peroxidases (POD) activity was determined using the oxidation rate of guaiacol at 470 nm with H_2_O_2_ according to the method described by Zhang et al. (2015). Catalase (CAT) activity was determined based on the rate of decrease in the absorbance of H_2_O_2_ at 240 nm (Qiu et al., 2011). Ascorbate peroxidase (APX) activity was quantified as the decrease in the absorbance of ascorbic acid at 290 nm (Wei et al., 2021).

The ascorbic acid (AsA) content was determined according the methods described by Chen et al. (2022): fresh samples (0.5 g) were homogenized in 10 mL of ice-cold 5% phosphoric acid and centrifuged at 10,000 g for 10 min at 4 °C. The reaction mixture consisted of 0.5 mL of 0.4% H_3_PO_4_-ethanol, one mL of 0.5% 4,7-diphenyl-1,10-phenanthroline-ethanol, 0.5mL of 0.03% FeCl_3_-ethanol, and then placed at 30 °C for 90 min. The absorbance was measured at 534 nm, and AsA content was calculated from the standard curve of ascorbate.

Glutathione (GSH) content was determined according to the method used by Yan et al. (2021): frozen fresh samples (0.5 g) were triturated in 5% trichloroacetic acid, centrifuged at 10,000 g for 10 min, and the supernatant was used to determine GSH content. The supernatant (0.2 mL) was added to 2.6 mL of NaH_2_PO_4_ (pH 7.7) and 0.2 mL of 5,5-dithiobis (2-nitrobenzoic; DTNB). The absorbance was measured at 412 nm after 10 min at 30 °C, and the content of reduced GSH was calculated according to the standard curve.

The soluble protein content was measured according to the methods described by Kučerová et al. (2019): passed through Coomassie brilliant blue G-250 dye using bovine serum albumin (BSA) as a protein standard. Fresh leaf and panicle samples (0.5 g) were homogenized with 10mL Na phosphate buffer (pH 7.8) and then centrifuged at 10,000 g for 10 min. Supernatants and dye were pipetted in spectrophotometer cuvettes, and the absorbances were measured at 595 nm. Proline content was determined using a modified method (Shabnam et al., 2016). Fresh leaf and panicle samples (0.3 g) were homogenized in 10 mL of 80% ethanol and then centrifuged at 10,000 g for 10 min. The supernatant (two mL) was mixed with four mL of 1.25% acid ninhydrin in a 100 °C water bath for 30 min, and the absorbance was measured at 508 nm, after cooling. The content of the proline was calculated according to the standard curve.

### Determination of panicle traits and yield components

At the mature stage, representative rice plants were selected according to the average number of panicles. These rice plants were then air-dried and tested indoors. The length of the internode under the main stem was measured, the panicle was weighed, the panicle length was measured, and the number of primary branches and secondary branches was recorded. After manual threshing and drying, the saturated grains and empty grains were separated by air flow and then measured. The saturated grains and the empty grains were weighed, and the seed setting rate per plant, 1,000-grain weight, and yield per plant were calculated.

### Statistical analysis

The data were processed using Excel (2019; Microsoft Corp., Redmond, WA, USA) and were analyzed using one-way ANOVA and the Duncan multiple comparison method using SPSS (25.0; IBM Corp., Armonk, NY, USA). Origin 2018 was used for drawing.

## Results

### Effects of Pro-Ca on the morphological indexes of rice under salt stress

Salt stress limited the growth of both cultivars of rice plants tested (Table S1). Compared with the control plants, the plant height, leaf area, and dry weight of the shoot of both HHZ and XLY900 decreased under NaCl treatment. The plant height decreased significantly by 5.66% (HHZ) and 4.63% (XLY900), and the leaf area decreased by 25.53% and 18.09%, respectively. The dry weight of the shoot decreased by 18.57% and 13.52%, respectively. Pro-Ca treatment significantly reduced the plant height of both cultivars but had no significant effect on leaf area and dry weight of the shoot. The phenotype and shoot biomass results demonstrated that the inhibition of rice growth induced by salinity stress was alleviated after foliar application with Pro-Ca. The leaf area of both HHZ and XLY900 significantly increased by 30.44% and 15.83% under treatment of Pro-Ca + NaCl as compared to the treatment of NaCl, respectively. These results confirmed that spraying Pro-Ca could regulate the growth of rice shoots under salt stress to a certain extent and alleviate the inhibition effect of salt stress on the rice plants (Fig. S1). The alleviation effects of Pro-Ca on HHZ were more substantial than on XLY900.

### Effects of Pro-Ca on the photosynthetic characteristics of rice under salt stress

**Figure 1:**
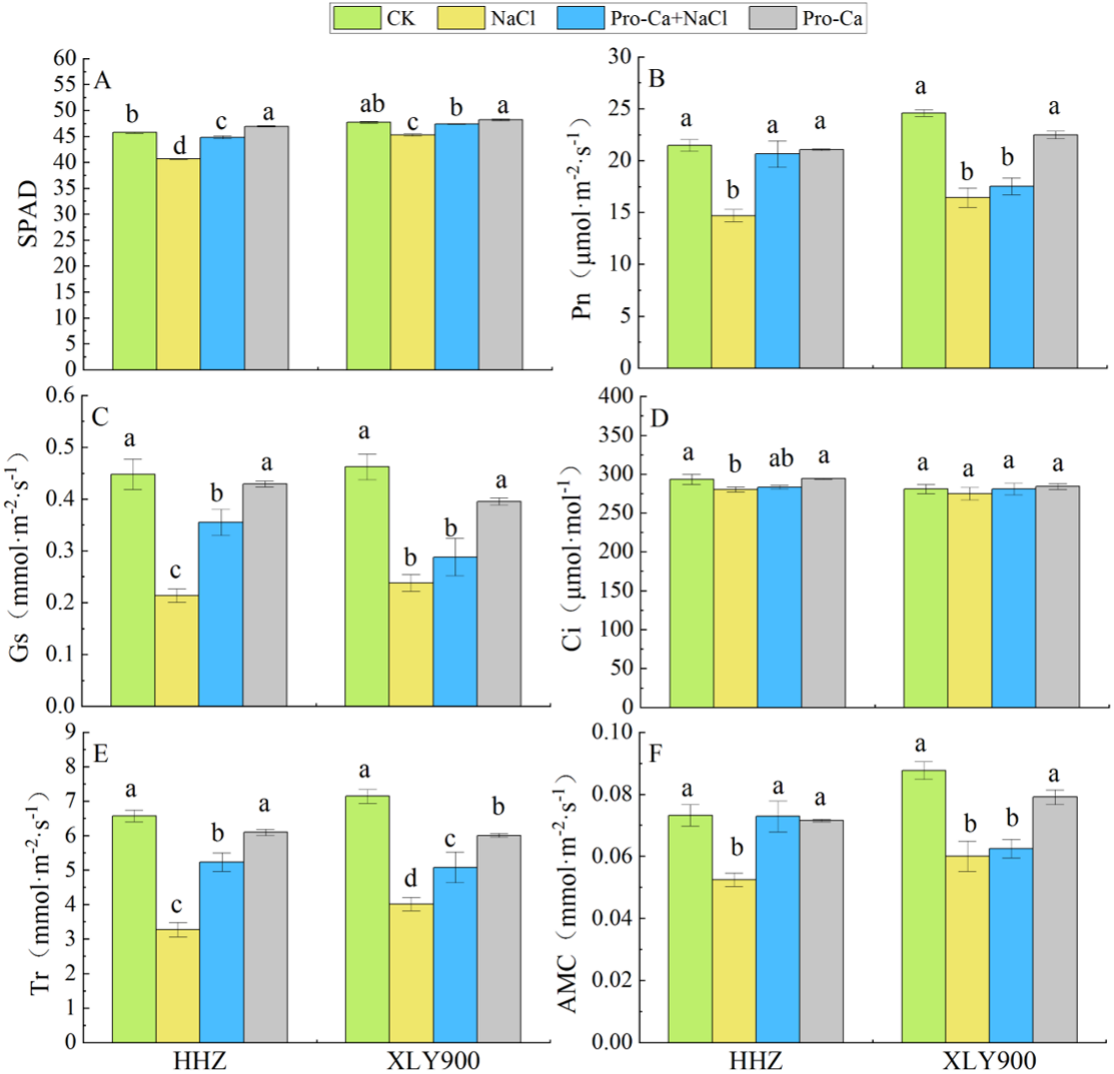
Effects of Pro-Ca spraying at booting stage on SPAD and photosynthetic parameters of rice under salt stress. (A) SPAD. (B) Net photosynthetic rate (Pn). (C) Stomatal conductance (Gs). (D) Intracellular CO_2_ concentrations (Ci). (E) Transpiration rate (Tr). (F) Apparent mesophyll conductance (AMC). Notes: HHZ: *Huanghuazhan* rice cultivars. XLY900: *Xiangliangyou900* rice cultivars. Different lowercase letters indicate that the mean values of the replicates were significantly different among the treatments (*p* < 0.05).

The SPAD values of HHZ and XLY900 significantly decreased under NaCl treatment, by 11.14% and 5.10%, respectively, compared with the control plants. In contrast, Pro-Ca + NaCl treatment increased the SPAD values by 10.26% (HHZ) and 4.64% (XLY900), compared with NaCl treatment alone (Fig. 1A). The Pn, Gs, Ci, Tr, and AMC of HHZ and XLY900 showed the variation trends under different treatments (Figs. 1B, 1C, 1D, 1E and 1F): NaCl stress significantly decreased Pn, Gs, Tr, and AMC in the two rice cultivars compared with the control plants. Reductions in Pn, Gs, Tr, and AMC in the HHZ cultivar were 31.52%, 52.23%, 50.28%, and 28.38%, respectively. In the XLY900 cultivar, these reductions were 33.20%, 48.52%, 43.74%, and 31.55%, respectively. The Ci of the two rice cultivars also decreased, but only significantly in the HHZ cultivar.

The combined treatment of Pro-Ca and NaCl alleviated the inhibitory effect of salt stress on the photosystem. It also significantly increased the Pn, Gs, Tr, and AMC in the HHZ cultivar compared with NaCl treatment alone (36%, 65.89%, 60.04%, and 38.95%, respectively). In the XLY900 cultivar, these parameters also increased by 6.70%, 21.01%, 26.47%, and 4.08%, respectively, but these increases were not significant except Tr. These results showed that salt stress inhibited the synthesis of chlorophyll and the progress of photosynthesis, and that Pro-Ca could alleviate this inhibition, maintaining a high photosynthetic efficiency in rice leaves under salt stress.

### Effects of Pro-Ca on osmotic regulators in rice leaves and panicles under salt stress

**Figure 2:**
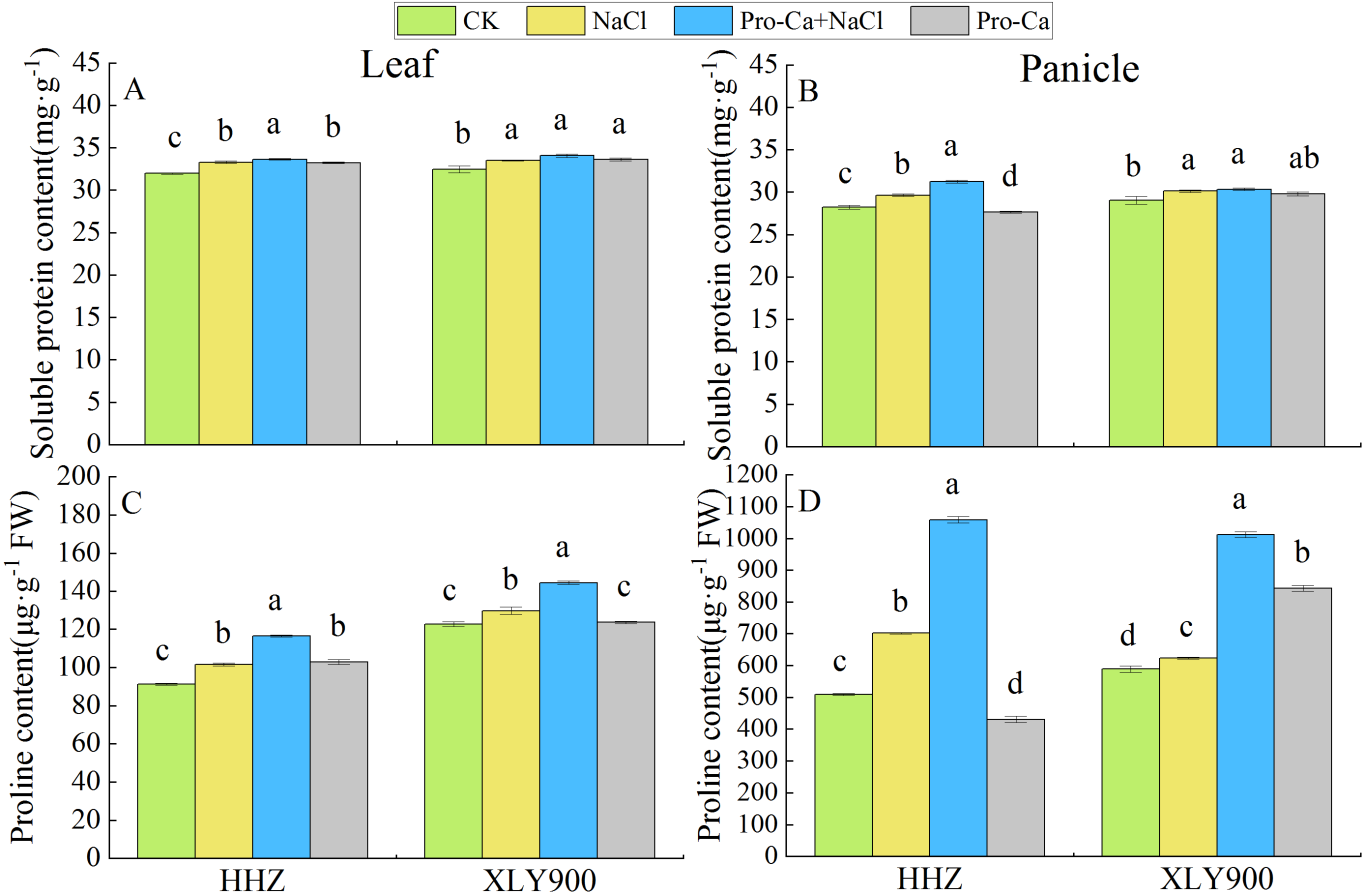
Effects of spraying Pro-Ca at the booting stage on soluble protein and proline content of rice leaves and panicles under salt stress. Changes in the soluble protein and proline content of leaves (A, C) and panicles (B, D) of *Huanghuazhan* (HHZ) and *Xiangliangyou900* (XLY900) rice cultivars in each of the four treatment groups: distilled water (control, CK); 0.3% NaCl (NaCl); Pro-Ca + 0.3% NaCl (Pro-Ca + NaCl); and Pro-Ca (Pro-Ca). Different lowercase letters indicate that the mean values of the replicates were significantly different among the treatments (*p* < 0.05).

The osmotic adjustment substance displayed significant differences between the leaves and panicles. For example, the proline content was remarkably higher in the panicles of the control plants than in the leaves (Figs. 2C and 2D). The soluble protein content of the leaves and panicles under NaCl treatment were significantly higher in both HHZ and XLY900 than in the control plants (Figs. 2A and 2B). Foliar spraying of Pro-Ca under salt stress significantly increased the soluble protein content of the leaves and panicles of HHZ, by 1.01% and 5.39%, respectively, compared with NaCl stress, but had no significant impact on the soluble protein content of XLY900 (Figs. 2A and 2B). Compared with control plants, the proline content of the leaves and panicles of both rice cultivars significantly increased after both NaCl treatment alone and after Pro-Ca + NaCl treatment (Figs. 2C and 2D). After NaCl treatment, compared with control plants, the proline content of HHZ increased by 11.25% in the leaves and 38.06% in the panicles, and the proline content of XLY900 increased by 5.68% in the leaves and 5.90% in the panicles. After Pro-Ca + NaCl treatment, the proline content was 14.61% higher in the HHZ leaves and 50.85% higher in the HHZ panicles than after NaCl treatment alone. In XLY900, these increases were 11.26% in the leaves and 62.44% in the panicles (Figs. 2C and 2D), indicating that foliar spraying of Pro-Ca could promote the biosynthesis of osmotic adjustment substances in rice leaves and panicles under salt stress, thereby maintaining osmotic pressure in the cells.

### Effects of Pro-Ca on the activity levels of antioxidant enzymes in rice leaves and panicles under salt stress

**Figure 3:**
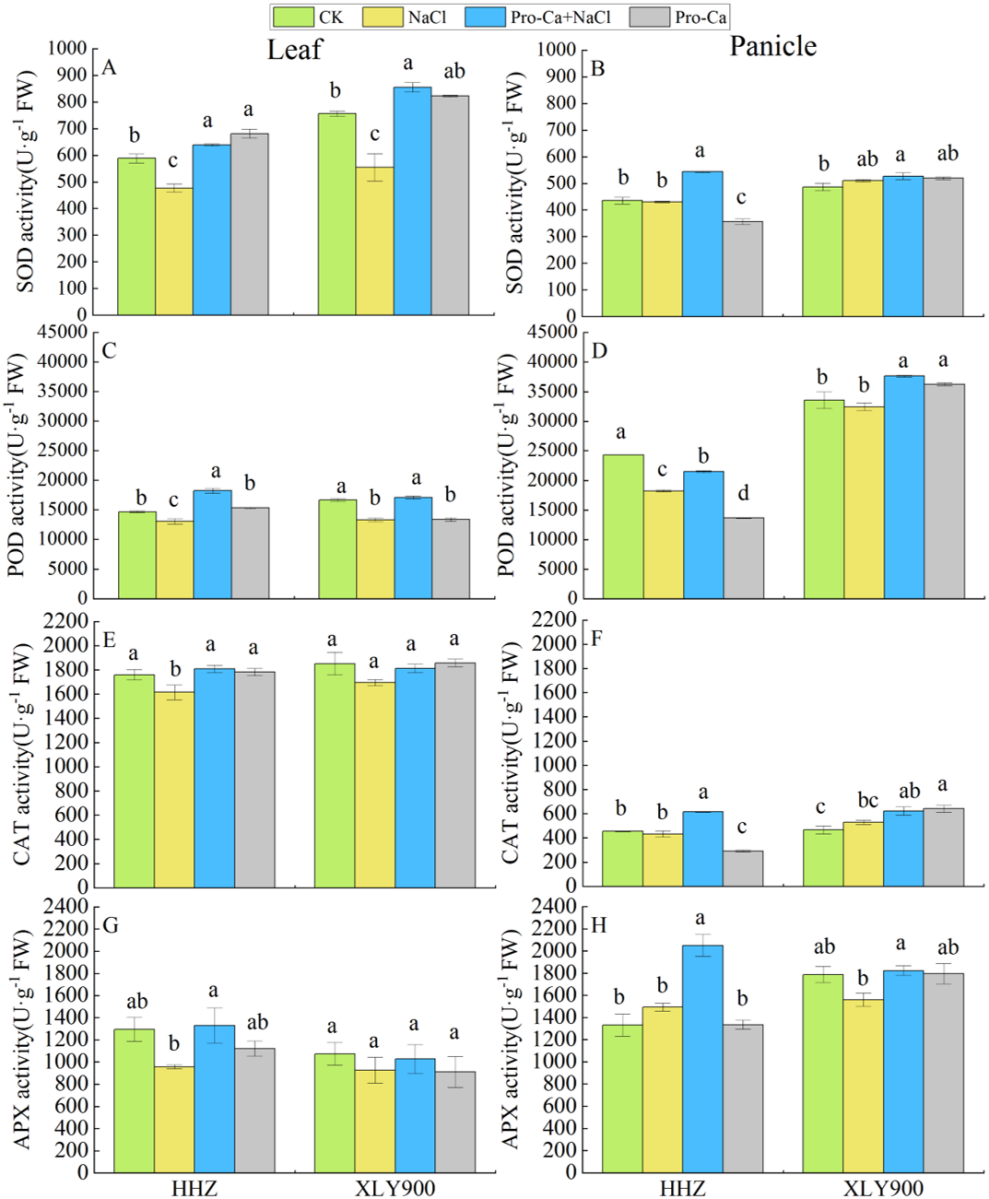
Effects of Pro-Ca spraying at the booting stage on the activities of antioxidant enzymes of rice leaves and panicles under salt stress. Changes in the activity levels of SOD, POD, CAT, APX in the leaves (A, C, E, G) and panicles (B, D, F, H) of *Huanghuazhan* (HHZ) and *Xiangliangyou900* (XLY900) rice cultivars in each of the four treatment groups: distilled water (control, CK); 0.3% NaCl (NaCl); Pro-Ca + 0.3% NaCl (Pro-Ca + NaCl); and Pro-Ca (Pro-Ca). Different lowercase letters indicate that the mean values of the replicates were significantly different among the treatments (*p* < 0.05).

The activity levels of SOD, POD, CAT, and APX in the leaves of HHZ and XLY900 were all inhibited under NaCl treatment, with SOD activity inhibited the most significantly, decreasing 18.92% in HHZ and 26.62% in XLY900. POD activity decreased the second most, by 11.04% in HHZ and 19.99% in XLY900 (Figs. 3A, 3C, 3E and 3G). The activity levels of all the antioxidant enzymes measured in the rice leaves were affected by salt stress, but the activity levels of SOD, POD, CAT, and APX in the rice panicles varied differently in response to salt stress. The activity level of POD in the panicle of HHZ was significantly lower (24.98%) than the control (Figs. 3B, 3D, 3F and 3H). Spraying Pro-Ca significantly enhanced the activity levels of SOD, POD, CAT, and APX enzymes in NaCl-treated HHZ leaves by 33.90%, 39.58%, 12.03%, and 38.86%, respectively and in NaCl-treated HHZ panicles by 26.26%, 17.74%, 42.30%, and 37.37%, respectively. Conversely, in NaCl-treated XLY900, the activity levels of SOD, POD, CAT, and APX all increased after Pro-Ca treatment, but only changes in the SOD and POD activity levels in XLY900 leaves reached significance, increasing by 54.21% and 28.36%, respectively, and only POD and APX activity levels in XLY900 panicles changed significantly, increasing by 15.98% and 17.01%, respectively. These results demonstrated that salt stress destroyed the antioxidant enzyme system in rice leaves and panicles, and spraying Pro-Ca under salt stress conditions improved the activity of antioxidant enzymes. In general, Pro-Ca had a larger impact on the activity levels of antioxidant enzymes in HHZ.

### Effects of Pro-Ca on AsA and GSH content in leaves and panicles of rice under salt stress

**Figure 4:**
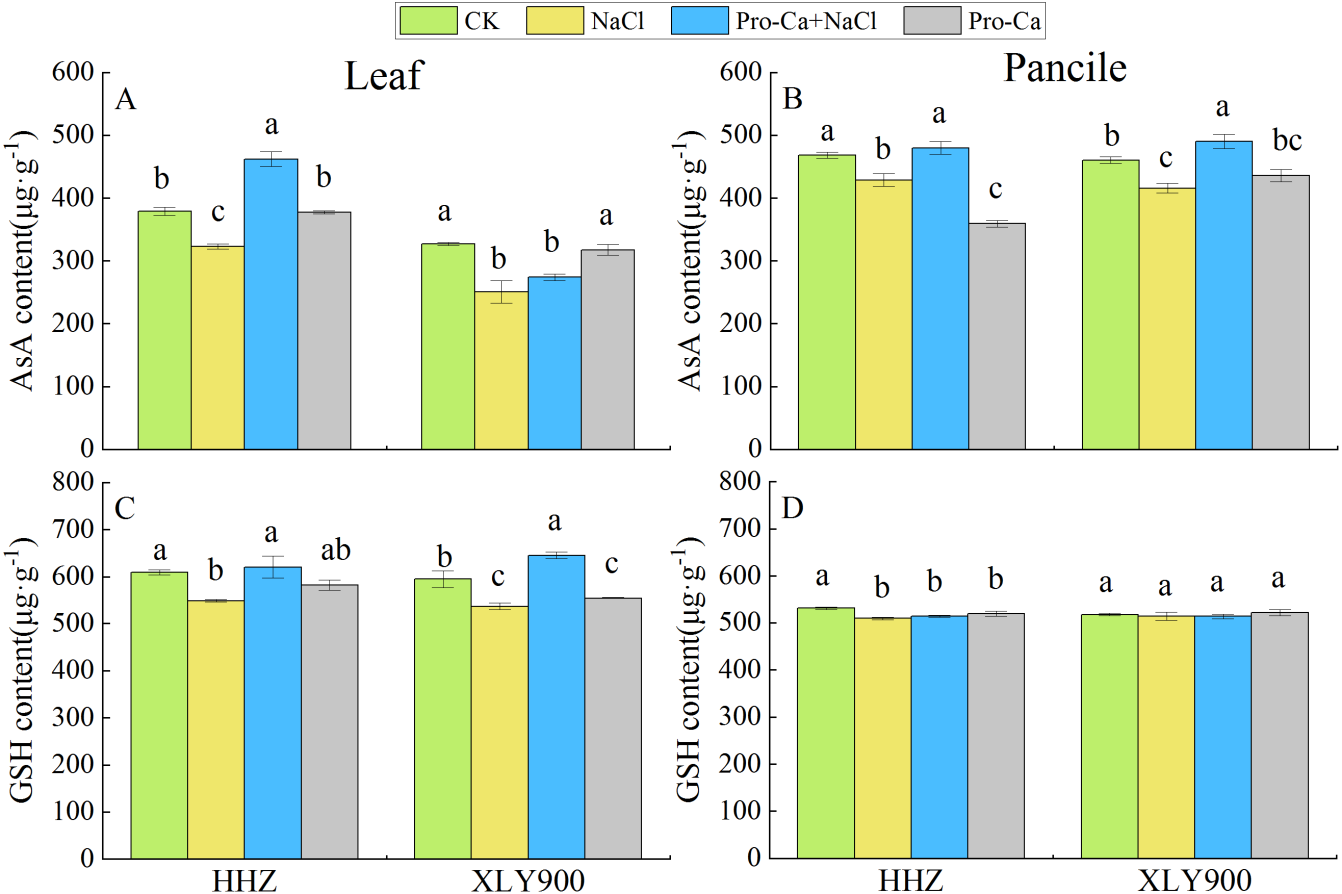
Effects of Pro-Ca spraying at the booting stage on AsA content and GSH content of rice leaves and panicles under salt stress. Changes in the AsA content (A, B) and GSH content (C, D) of the leaves and panicles of *Huanghuazhan* (HHZ) and *Xiangliangyou900* (XLY900) rice cultivars in each of the four treatment groups: distilled water (control, CK); 0.3% NaCl (NaCl); Pro-Ca + 0.3% NaCl (Pro-Ca + NaCl); and Pro-Ca (Pro-Ca). Different lowercase letters indicate that the mean values of the replicates were significantly different among the treatments (*p* < 0.05).

NaCl treatment significantly reduced AsA content in the leaves and panicles of both HHZ and XLY900 cultivars, compared to controls (Figs. 4A and 4B). The GSH content (Figs. 4C and 4D) also decreased significantly in both HHZ and XLY900 cultivars, but the decrease in GSH content in the panicles of XLY900 did not reach a significance. Compared with NaCl treatment, Pro-Ca + NaCl treatment increased AsA and GSH content in the leaves of both rice cultivars, with AsA and GSH significantly increasing by 43.07% and 13.11% in HHZ leaves, respectively. In XLY900 leaves, AsA content increasing by 9.20% and GSH content increasing by 20.24%, but the increase in AsA content was not significant. Spraying Pro-Ca significantly enhanced the AsA content of both cultivars in NaCl-treated panicles with AsA increasing 11.87% in NaCl-treated HHZ panicles and 17.87% in NaCl-treated XLY900 panicles. These results indicated that spraying Pro-Ca could reverse decreases in antioxidant content in rice caused by salt stress.

### Effects of Pro-Ca on internode length and panicle traits in rice under salt stress

**Figure 5:**
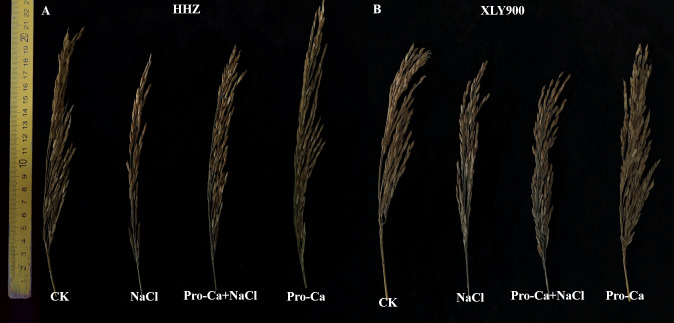
The images of *Huanghuazhan* (HHZ) and *Xiangliangyou900* (XLY900) rice cultivars panicle traits under salt stress by spraying Pro-Ca at the booting stage. The left half is HHZ (A), and the right half is XLY900 (B). The two rice cultivars from left to right were treated as follows: distilled water (control, CK); 0.3% NaCl (NaCl); Pro-Ca + 0.3% NaCl (Pro-Ca + NaCl); Pro-Ca (Pro-Ca).

Figure 5 shows the panicle traits of HHZ and XLY900 at maturity under different treatments. The results show that salt stress was not conducive to the growth of rice panicles. NaCl treatment significantly affected the uppermost internode length, panicle length, panicle weight, and the number of primary branches of both HHZ and XLY900 (Table 1). In the HHZ cultivar, the reductions in the uppermost internode length, panicle length, panicle weight, and the number of primary branches were 20.64%, 6.29%, 41.99%, and 14.53%, respectively. In the XLY900 cultivar, these reductions were 25.41%, 8.34%, 36.19%, and 8.99%, respectively. Compared with the control plants, the number of secondary branches of both cultivars showed a slight change after NaCl treatment or Pro-Ca + NaCl treatment. Furthermore, spraying Pro-Ca under NaCl stress significantly enhanced the uppermost internode length, panicle length, panicle weight, and the number of primary branches, but not the number of secondary branches, compared to NaCl treatment alone (Table 1). In HHZ, Pro-Ca increased the panicle length, panicle weight, and the number of primary branches by 12.15%, 78.05%, and 18.17%, respectively. In XLY900, Pro-Ca increased the panicle length, panicle weight, and the number of primary branches by 8.08%, 30.81%, and 9.02%, respectively. In general, Pro-Ca had a larger impact on the internode length and panicle traits of HHZ than XLY900. These results also show that spraying Pro-Ca could alleviate the effects of salt stress on panicle development.

**Table 1:** Effects of Pro-Ca spraying at the booting stage on internode length and panicle traits of rice under salt stress. The data in the table are the mean ± standard error (SE), and different lowercase letters in the same column indicate that the mean values of the replicates were significantly different among the treatments (*p* < 0.05).

Variety	Treatment	Uppermost internode length (cm)	Panicle length (cm)	Panicle weight (g)	Number of primary branches	Number of secondary branches
HHZ	CK	37.12 ± 0.59a	20.11 ± 0.36b	9.27 ± 0.32a	12.16 ± 0.13a	17.36 ± 1.57ab
	NaCl	29.46 ± 0.72c	18.84 ± 0.22c	5.38 ± 0.45b	10.39 ± 0.20b	13.80 ± 2.19b
	Pro-Ca+ NaCl	32.85 ± 0.55b	21.13 ± 0.16a	9.57 ± 0.66a	12.28 ± 0.21a	19.34 ± 1.99ab
	Pro-Ca	34.69 ± 0.92b	20.59 ± 0.40ab	10.55 ± 0.43a	11.68 ± 0.31a	21.88 ± 1.82a
XLY900	CK	34.83 ± 0.91a	19.80 ± 0.20a	10.94 ± 0.67a	13.70 ± 0.22a	31.92 ± 1.33a
	NaCl	25.98 ± 0.68c	18.15 ± 0.19b	6.98 ± 0.41c	12.47 ± 0.34b	29.43 ± 1.50a
	Pro-Ca+ NaCl	31.70 ± 0.96b	19.62 ± 0.21a	9.13 ± 0.68b	13.59 ± 0.13a	32.62 ± 2.00a
	Pro-Ca	32.79 ± 0.77ab	19.85 ± 0.23a	8.74 ± 0.66bc	13.51 ± 0.24a	31.79 ± 2.02a

**Notes.**

HHZ Huanghuazhan rice cultivars XLY900 Xiangliangyou900 rice cultivars

### Effects of Pro-Ca on rice yield and its components under salt stress

As shown in Table 2, NaCl stress significantly decreased the grain yield and the following rice yield components: grains per panicle, seed setting rate, and 1,000-grain weight. In general, the reductions caused by NaCl stress were less severe in XLY900. In HHZ, the reductions in the grains per panicle, seed setting rate, 1,000-grain weight, and grain yield per plant were 30.11%, 39.43%, 10.47%, and 46.03%, respectively, while in XLY900, they were 23.62%, 20.16%, 9.04%, and 41.38%, respectively. Pro-Ca mitigated these inhibitions to a different extent than NaCl treatment. The increase in the grains per panicle, seed setting rate, 1,000-grain weight and grain yield of per plant in HHZ after Pro-Ca treatment were 55.73%, 62.25%, 3.21%, and 94.81%, respectively, with the changes in grains per panicle, seed setting rate, and grain yield per plant reaching significance. In XLY900 after Pro-Ca treatment, the grains per panicle, seed setting rate, 1,000-grain weight, and grain yield of per plant increased by 28.59%, 17.93%, 2.35%, and 48.97%, respectively, with only the mitigation effect on yield reaching a significant level. This indicates that Pro-Ca alleviated the effects of salt stress on rice yield and its components, with HHZ seeing a larger alleviation effect of Pro-Ca than XLY900.

**Table 2:** Effects of Pro-Ca spraying at the booting stage on rice yield and its constituent factors under salt stress. The data in the table are the mean ± standard error (SE), and different lowercase letters in the same column indicate that the mean values of the replicates were significantly different among the treatments (*p* < 0.05).

Cultivar	Treatment	Grain number per panicle	Seed setting rate	Thousand grains weight (g)	Yield (g/plant)
HHZ	CK	407.20 ± 17.00a	0.85 ± 0.04a	24.52 ± 0.53a	8.49 ± 0.38a
	NaCl	284.60 ± 29.86b	0.52 ± 0.02b	21.95 ± 0.41b	4.58 ± 0.23b
	Pro-Ca+ NaCl	443.20 ± 31.12a	0.85 ± 0.01a	22.66 ± 0.48b	8.93 ± 0.86a
	Pro-Ca	480.20 ± 30.72a	0.79 ± 0.02a	25.35 ± 0.42a	9.61 ± 0.65a
XLY900	CK	517.40 ± 45.91a	0.73 ± 0.02a	27.11 ± 0.36a	9.63 ± 0.71a
	NaCl	395.20 ± 29.69a	0.58 ± 0.04b	24.66 ± 0.39b	5.64 ± 0.50b
	Pro-Ca+ NaCl	508.20 ± 11.08a	0.69 ± 0.03ab	25.24 ± 0.62b	8.41 ± 1.10a
	Pro-Ca	441.60 ± 59.91a	0.66 ± 0.05ab	25.25 ± 0.46b	8.20 ± 0.91a

**Notes.**

HHZ Huanghuazhan rice cultivars XLY900 Xiangliangyou900 rice cultivars

## Discussion

### Pro-Ca improved the growth and photosynthetic capacity of rice under salt stress

Salt stress is one of the main adversities affecting the growth and development of crops. Different plants have different responses to salt stress, and even different varieties, growth stages, and organs of the same plant can respond differently to salt stress (Singh et al., 2019; Chang et al., 2019; Frukh et al., 2020; Gerona et al., 2019; Liu et al., 2019). Shoot is an essential metabolic and synthetic organ, shoot growth can reflect the salt tolerance of plants, with more shoot growth indicating a higher salt tolerance. Previous studies have shown that salt stress can change the morphological growth of rice, causing decreases in plant height and biomass, and a stop increasing leaf area (Hussain et al., 2013). This study found that in the booting stage of rice, the plant height, leaf area, and dry weight of the shoot were all lower than controls under salt stress, with HHZ cultivars seeing larger decreases than XLY900 cultivars (Table S1). Spraying exogenous Pro-Ca significantly alleviated NaCl-inhibited leaf areas, especially in HHZ cultivars. This finding aligns with the results of Wang et al. (2020), who found that rice cultivars that were more sensitive to stress were also more affected by plant growth regulators.

Photosynthesis is crucial to plant growth and yield. All components involved in the photosynthetic reactions may be affected by salinity, including photosynthetic pigments, photosystems, gas exchange processes, and enzymes involved in carbon metabolism (Ashraf & Harris, 2013). Gadelha et al. (2021) reported that the chloroplast membrane system of plants were damaged to a certain extent when exposed to salt stress, resulting in the decomposition of chloroplasts and ultimately the decline of the net photosynthetic rate. In this study, the SPAD, Pn, Gs, Tr, and AMC of rice leaves were significantly lower under salt stress (Figs. 1A, 1B, 1C, 1E and 1F), indicating that a salt stress environment may destroy the chloroplast of rice leaves, hinder the synthesis of chlorophyll, or accelerate the degradation of pigments, and hinder the efficiency of water and light energy use in leaves, resulting in decreased photosynthetic activity (Qu et al., 2012). Furthermore, the decrease of Ci in salt-treated rice leaves was accompanied by the decrease of Gs in this study, indicating that the main reason for the decrease of Pn may be stomatal limitation (Pan et al., 2020), which was similar to the results of Guo et al. (2018). The decrease of Pn might also be due to the decrease in the activity levels of enzymes involved in the photosynthetic process, such as the Rubisco enzyme, sucrose phosphate synthase, and nitrate reductase in leaves (Ashraf & Harris, 2013). The present study also found that the intervention of exogenous Pro-Ca under salt stress conditions significantly increased chlorophyll content in both HHZ and XLY900 cultivars compared with the salt treatment alone (Fig. 1A), which may be due to Pro-Ca’s ability to protect the thylakoid membranes and slow down the degradation of chlorophyll (Feng et al., 2021). Foliar spraying of Pro-Ca increased the Pn, Gs, Ci, and AMC of both HHZ and XLY900 cultivars under salt stress, indicating that Pro-Ca could enhance photosynthesis. This may be because Pro-Ca induces the stomatal opening, which affects the diffusion process of CO_2_ in rice leaves, increasing CO_2_ diffused from the external environment to mesophyll tissue through the stomatal opening, thereby increasing the rate of photosynthetic carbon assimilation. The above results indicate that Pro-Ca could maintain the chloroplast structure and improve the photosynthetic capacity of rice, thereby alleviating the inhibitory effect of salt stress on rice growth.

### Pro-Ca modulated osmotic stress under salt stress

Salt stress can induce osmotic stress in plants, limiting water uptake and causing physiological drought (Liu et al., 2022). As an important osmoprotectant, proline is essential in protecting subcellular structure and osmoregulation under stress conditions, and is also a critical indicator of a plant’s salt tolerance (Gerona et al., 2019). Ahmad et al. (2016) found that the soluble protein and proline content of chickpeas increased under high-salt conditions to resist salt stress. Gerona et al. (2019) demonstrated that salt stress significantly increased proline content in rice leaves and panicles at the flowering stage, improving rice tolerance to salt stress. In this study, the contents of soluble protein and proline in the leaves and panicles of rice plants under salt stress at the flowering stage were significantly higher than control plants (Fig. 2), and the accumulation of proline was relatively high (Fig. 2D), indicating that under salt stress, rice could generate a self-protection mechanism by synthesizing osmotic adjustment substances in different parts of the plant (Liu et al., 2022). Proline is an amino acid that can provide nutrition for the reproductive stage of plants, helping with pollen development and dissemination (Biancucci et al., 2015), so the considerable accumulation of proline seen in the panicles in this study may be due to its role in stabilizing proteins and providing nutrition in the reproductive stage (Schmidt, Situ & Ulmer, 2016). Previous studies have shown that exogenous Pro-Ca can induce proline accumulation in plants under stress (Soleimani Aghdam, 2013; Ozbay & Susluoglu, 2016). The results of this study found that spraying Pro-Ca at the booting stage further increased the soluble protein and proline content of leaves and panicles compared with salt treatment, indicating that Pro-Ca could induce the accumulation osmolytes in the leaves and panicles of rice plants, better protecting them from damage caused by salt stress. This result also confirms that the application of Pro-Ca could increase the content of osmolytes in rice leaves, thereby improving the stomatal conductance of rice leaves under salt stress and slowing down the inhibition of photosynthetic efficiency caused by salt stress (Ashraf & Harris, 2013).

### Pro-Ca activated the antioxidant defense system under salt stress

Plants possess antioxidant defense systems to eliminate ROS from salt stress, including antioxidant enzymes and non-enzymatic antioxidants, such as SOD, POD, CAT, APX, GSH and AsA (Hossain & Dietz, 2016; Singh & Flowers, 2016). This study found that the activity levels of SOD, POD, CAT, and APX in the leaves of the two rice varieties tested decreased under salt stress (Fig. 3), indicating that the antioxidant enzyme system of leaves was destroyed by salt concentration in this study. However, the antioxidant enzyme activity of XLY900 was relatively stable, similar to the results Qiu et al. (2014) found in wheat. Our results also showed that the activity levels of the antioxidant enzymes in rice leaves and panicles were different, which may be due to differences in the sensitivity of rice vegetative and reproductive tissues to sodium ions or changes in sodium ion movement in different parts of the plant (Hasegawa, 2013; Gerona et al., 2019; Chakraborty et al., 2019), resulting in different responses in the antioxidant systems of the leaf and panicle. Cheng et al. (2019) applied thymol to rice under salt stress by irrigation. They found that the expression levels of *SOD*, *CAT*, and *APX*-related genes in the roots were up-regulated, thereby enhancing the activity levels of antioxidant enzymes in rice roots to resist salt stress. In this study, foliar spraying of Pro-Ca significantly increased the activity levels of SOD and POD in the leaves and the activity levels of POD and APX in the panicles (Figs. 3A, 3C, 3D and 3H). These results indicate that spraying Pro-Ca on leaves may up-regulate the expression levels of genes encoding *SOD*, *POD*, and *APX* in the leaves and panicles, improving enzyme activity, but this hypothesis needs to be further explored in transcriptomics. This study also found that AsA and GSH content in the leaves and panicles of both HHZ and XLY900 decreased under salt stress (Fig. 4), which may be because AsA and GSH participate in the process of scavenging oxygen free radicals under salt stress. Moreover, Pro-Ca application increased the AsA content of NaCl-treated leaves and panicles, so Pro-Ca may eliminate H_2_O_2_ by increasing AsA content and improving the AsA-GSH cycle to help stop the oxidative damage caused by salinity. The increase in APX activity seen in this study was also conducive to the AsA-GSH cycle (Fig. 3G). In addition, AsA has antioxidant effects and affects critical enzymatic reactions; since it is a cofactor for some enzymes (Bielen et al., 2013), more studies may be needed to determine the role of AsA in different organs of rice exposed to salt stress.

### Pro-Ca enhanced rice yield and panicle traits under salt stress

The booting stage is a critical period for determining the development or degeneration of spikelets. Previous studies have shown that stress at the booting stage of rice will affect panicle and yield traits, such as causing a significant decrease in panicle length, number of branches, and grain size, which ultimately affects the 1,000-grain weight and total yield of rice (Zhu, Hu & Zhu, 2011; Gerona et al., 2019; Wang et al., 2020). This study found that the panicle length, panicle weight, number of primary branches, 1,000-grain weight, and yield per plant significantly decreased at the booting stage in both HHZ and XLY900 cultivars under salt stress (Tables 1 and 2). Most of these traits decreased more significantly in HHZ, indicating that HHZ is more sensitive to the salt environment than XLY900 at the booting stage. Panicle and yield traits of rice were severely inhibited, similar to previous research results (Gerona et al., 2019). This may be due to the inhibition of root water uptake by salt stress at the reproductive stage, resulting in blocked physiological processes and unsatisfactory production and distribution of assimilates (Nasrudin, Isnaeni & Fahmi, 2022). It has been reported that Pro-Ca can shorten the length of internodes in rice and increase the seed setting rate, 1,000-grain weight, and yield of rice (Pal & Johal, 2019). In this study, the application of Pro-Ca significantly increased the panicle length, panicle weight, and the number of primary branches in rice plants under salt stress, indicating that Pro-Ca has the potential to protect rice panicle differentiation and development and increase the branch number of rice plants. In terms of yield and yield components, the number of grains per panicle, seed setting rate, and 1,000-grain weight are the main factors that constitute yield, and these three factors mutually restrict each other. This study found that the number of grains per panicle, seed setting rate, 1,000-grain weight, and yield of HHZ and XLY900 improved by varying degrees after the application of Pro-Ca compared with salt treatment, which may be because the application of exogenous Pro-Ca was conducive to the formation of pollen mother cells and reduced abortion, thereby improving the seed setting rate, and Pro-Ca may have the potential to control rice vegetative growth and increase the transportation of assimilates to grains, increasing rice yield (Lokuruge et al., 2015). These results indicate that salt stress hurts panicle traits, yield, and yield components of rice at the booting stage, while foliar spraying of Pro-Ca has a positive regulatory effect on rice plants under salt stress, which increases yield. However, the mechanism of Pro-Ca in regulating rice panicle traits under salt stress and the detailed molecular mechanism used to confer salt tolerance to rice plants still need to be further explored.

## Conclusions

In this study, the growth and photosynthetic data showed that salt stress inhibited the morphological, physiological, and metabolic processes, as well as the panicle, and yield traits of rice plants at the booting stage, and that spraying Pro-Ca could alleviate these impacts. The physiological data of different parts of the rice plant showed that Pro-Ca could endow rice with salt tolerance by activating the rice antioxidant defense system and enhancing osmotic adjustment ability. Moreover, the data at plant maturity showed that, compared with the single salt stress treatment, spraying Pro-Ca reduced the adverse effects of salt stress on the panicle traits, yield, and yield components of rice, thereby increasing rice yield under salt stress. Therefore, spraying Pro-Ca to improve salt stress and increase crop yield is a feasible chemical control solution for developing and utilizing saline-alkali land. Future research should focus on the detailed molecular mechanisms of rice plants impacted by Pro-Ca at the reproductive stage.

##  Supplemental Information

10.7717/peerj.14673/supp-1 Figure S1 The images of shoot phenotype of rice under salt stress by spraying Pro-Ca at the booting stage. The left half is HHZ (A), and the right half is XLY900 (B). The two rice cultivars from left to right were treated as follows: distilled water (control, CK); 0.3% NaCl (NaCl); Pro-Ca + 0.3% NaCl (Pro-Ca + NaCl); Pro-Ca (Pro-Ca).

10.7717/peerj.14673/supp-2 Table S1 Effects of Pro-Ca spraying at the booting stage on rice growth under salt stress The data in the table are the mean ± standard error (SE), and different lowercase letters in the same column indicate that the mean values of the replicates were significantly different among the treatments (*p* < 0.05). HHZ: *Huanghuazhan* rice cultivars. XLY900: *Xiangliangyou900* rice cultivars.

10.7717/peerj.14673/supp-3 Supplemental Information 3 Raw data for figures and tables The data in the table are the mean ± standard error (SE), and different lowercase letters in the same column indicate that the mean values of the replicates were significantly different among the treatments (*p* < 0.05). HHZ: Huanghuazhan rice cultivars. XLY900: Xiangliangyou900 rice cultivars.
The raw data showed differences in physiological indicators between the two rice cultivars under different treatments. These indicators were used for analysis to explore the mitigating effect of prohexadione-calcium on salt stress.

## Additional Information and Declarations

### Data Availability

The following information was supplied regarding data availability: The raw measurements are available in the Supplementary File.
