# Supplementary figures and images for: Effects of prohexadione calcium spraying during the booting stage on panicle traits, yield, and related physiological characteristics of rice under salt stress
Source: PeerJ. 2023 Jan 23;11:e14673. doi: 10.7717/peerj.14673 (PMC9879151; doi:10.7717/peerj.14673)

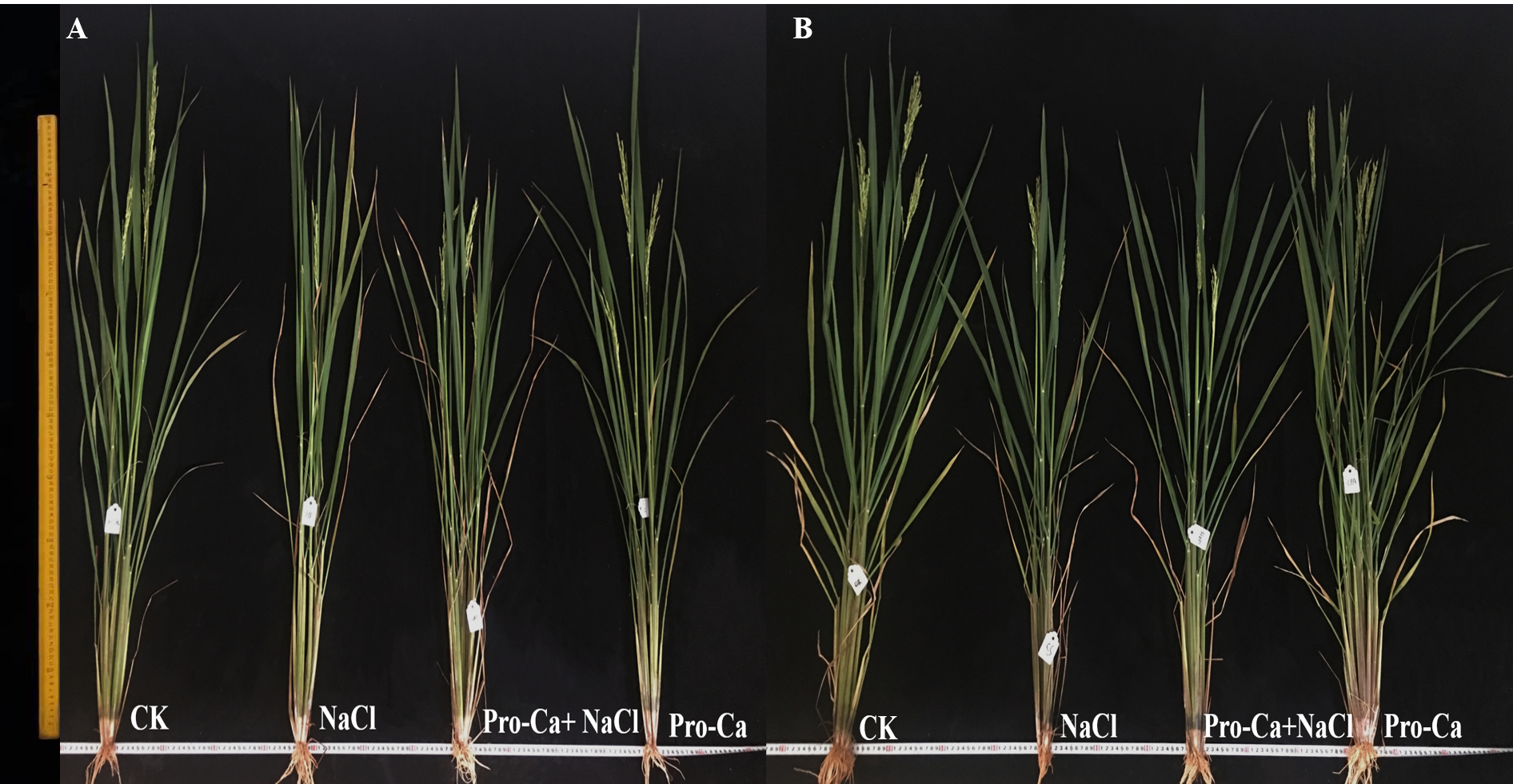

Supplement: Figure S1 — The left half is HHZ (A), and the right half is XLY900 (B). The two rice cultivars from left to right were treated as follows: distilled water (control, CK); 0.3% NaCl (NaCl); Pro-Ca + 0.3% NaCl (Pro-Ca + NaCl); Pro-Ca (Pro-Ca). [file peerj-11-14673-s001.png]
