# Supplementary material for: Effects of prohexadione calcium spraying during the booting stage on panicle traits, yield, and related physiological characteristics of rice under salt stress
Source: PeerJ. 2023 Jan 23;11:e14673. doi: 10.7717/peerj.14673 (PMC9879151; doi:10.7717/peerj.14673)
Supplement: Table S1 — The data in the table are the mean ± standard error (SE), and different lowercase letters in the same column indicate that the mean values of the replicates were significantly different among the treatments (p < 0.05). HHZ: Huanghuazhan rice cultivars. XLY900: Xiangliangyou900 rice cultivars. [file peerj-11-14673-s002.docx]

| Cultivar | Treatment | Plant height(cm) | Leaf area(cm²) | Shoot dry weight(g) |
| --- | --- | --- | --- | --- |
| HHZ | CK | 111.87±0.65a | 62.66±3.66a | 18.06±1.18a |
|  | NaCl | 105.53±0.79b | 46.67±0.26b | 14.71±1.58a |
|  | Pro-Ca + NaCl | 106.77±0.81b | 60.87±2.56a | 14.72±0.29a |
|  | Pro-Ca | 105.97±0.32b | 60.99±1.38a | 15.50±1.35a |
| XLY900 | CK | 112.23±0.86a | 64.34±2.08a | 19.04±0.96a |
|  | NaCl | 107.03±1.56b | 52.70±2.86b | 16.47±2.05a |
|  | Pro-Ca + NaCl | 108.27±1.03b | 61.05±1.18a | 17.10±2.07a |
|  | Pro-Ca | 108.33±0.88b | 64.27±1.09a | 20.01±2.49a |

**Table S1 Effects of Pro-Ca spraying at the booting stage on rice growth under salt stress.**
